# Supplementary material for: Scalable Production of Size-Controlled Cholangiocyte and Cholangiocarcinoma Organoids within Liver Extracellular Matrix-Containing Microcapsules
Source: Cells. 2022 Nov 18;11(22):3657. doi: 10.3390/cells11223657 (PMC9688401; doi:10.3390/cells11223657)
Supplement: Supplementary file 1 [file cells-11-03657-s001.zip › cells-1997805-supplementary.pdf]

# Scalable production of size-controlled cholangiocyte and cholangiocarcinoma organoids within liver extracellular matrix-containing microcapsules

**Gilles S. van Tienderen[1*], Jorke Willemse[1*], Bas van Loo[2*], Eline van Hengel[1], Jeroen de Jonge[1], Luc J.W. van der Laan[1], Jeroen C.H. Leijten[2] & Monique M.A. Verstegen[1]**

[1] Erasmus MC Transplant Institute, Department of Surgery, University Medical Center Rotterdam, the Netherlands

[2] Department of Developmental BioEngineering, Faculty of Science and Technology, Technical Medical Centre, University of Twente, Enschede, the Netherlands

[*] Shared first authorship

Correspondence: Monique MA Verstegen
Email address: m.verstegen@ermasmusmc.nl
Dr. Molewaterplein 40, Room Na1005
3015 GD, Rotterdam, The Netherlands
Phone: +31 10 703 5528

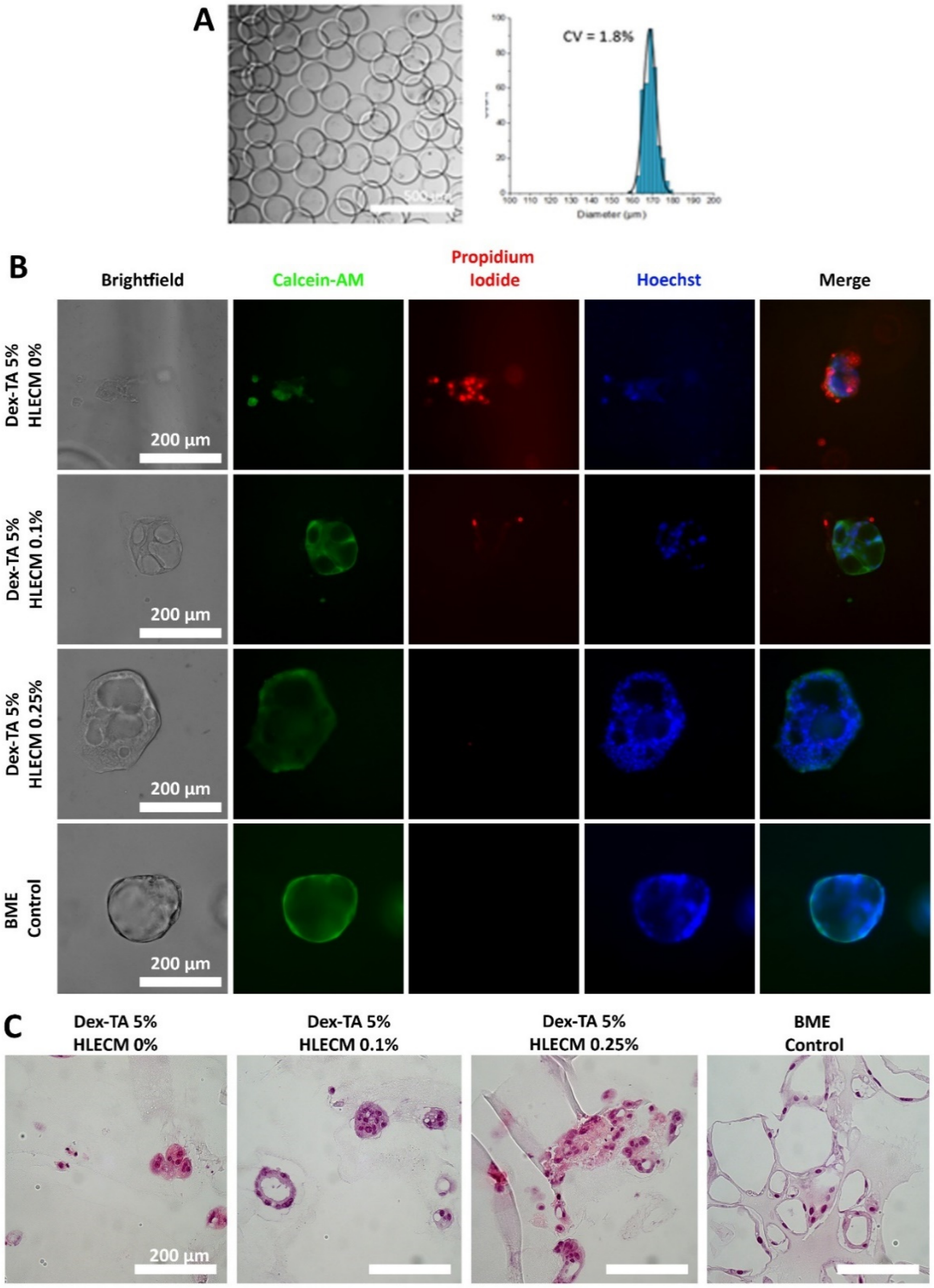


**Supplemental Figure S1: ICO can self-assemble within Dex-Ta microgel, but can only survive with the addition of HLECM**. **A)** Microcapsules can be produced with high monodispersity (CV=1.8%). **B)** Addition of HLECM to Dex-Ta increases cell viability, **C)** and allows for the self-organization of cells into organoid-like structures.

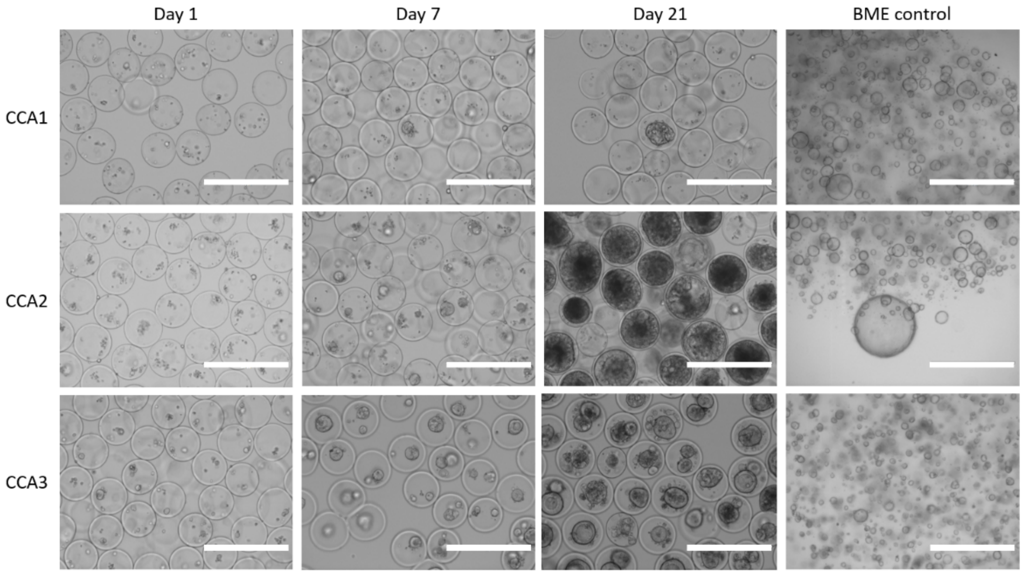


**Supplemental Figure S2. CCAO formation in microcapsules.** Representative bright field micrographs of CCAO cultured in BME control for 7 days or cultured in microcapsules for 1, 7, and 21 days. Scale bar indicates 400 µm.

**Table S1.** Medium components required for the formulation of basal medium (ADV+)

| Component | Amount | Concentration | Brand |
|---|---|---|---|
| Advanced DMEM/F12 | 500ml | | Gibco |
| HEPES | 5ml | 1M | Life technologies |
| L-Glutamin | 5ml | 100X | Life technologies |
| Primocin | 1ml | 500mg/ml | Invivogen |
| Pen/Strep | 5ml | 10000 U/ml | Life technologies |

**Table S2.** Medium components required for the formulation of Start Up Medium (SEM) or Expansion Medium (EM). Medium components with a * are only added to SEM.

| Component | Concentration | Brand |
|---|---|---|
| Adv+ | - | Gibco |
| N2 | 1% | Gibco |
| B27 | 2% | Gibco |
| N-Acetylcystein | 1mM | Sigma-Aldrich |
| gastrin | 10 nM | Sigma-Aldrich |
| EGF | 50 ng/ml | Peprotech |
| FGF10 | 100 ng/ml | Peprotech |
| HGF | 25 ng/ml | Peprotech |
| nicotinamide | 10nM | Sigma-Aldrich |
| A83.01 | 5 µM | Tocris |
| Forskolin | 10 µM | Torcris |
| R-Spondin | 10% | Conditioned medium |
| WNT* | 30% Wnt | Conditioned medium |
| Noggin* | 25 ng/ml | Conditioned medium |
| Y27632* | 10µM | Tocris |

| | | |
|---|---|---|
| hES cell cloning recovery solution* | 1:1000 dilution | Stemgent |

**Table S3**. List of primary antibodies used in this study

| Primary antibody | Raised in | Dilution | Supplier |
|---|---|---|---|
| ZO1 | Rabbit | 1:100 | Proteintech |
| KRT7 | Mouse | 1:100 | Dako |
| KRT19 | Mouse | 1:100 | Dako |
| KI67 | Rabbit | Ready –to-use | Kind gift from pathology |

**Table S4.** List of fluorescent labeled secondary antibodies used in this study

| Secondary antibody | Raised in | Against | Dilution | Supplier |
|---|---|---|---|---|
| Alexa 555 | Goat | Mouse | 1:100 | Fisher scientific |
| Alexa 488 | Goat | Rabbit | 1:100 | Fisher scientific |

**Table S5**. List of qPCR primers used in this study

| Primer | Species | Forward sequence 5'to 3' | Reverse sequence 5'to 3' |
|---|---|---|---|
| GAPDH | Human | CTTTTGCGTCGCCAGCCGAG | CCAGGCGCCCAATACGACCA |
| HPRT1 | Human | ACCAGTCAACAGGGGACATAA | CTTCGTGGGGTCCTTTTCACC |
| B2M | Human | GTGTCTGGGTTTCATCCATC | GGCAGGCATACTCATCTTTT |
| LGR5 | Human | GTCAGCTGCTCCCGAATCCC | TGAAACAGCTTGGGGGCACA |
| PROM1 | Human | CCTGGGGCTGCTGTTTATTA | ATCACCAACAGGGAGATTGC |
| KRT7 | Human | GGGGACGACCTCCGGAATAC | CTTGGCACGCTGGTTCTTGA |
| KRT19 | Human | GCACTACAGCCACTACTACACGA | CTCATGCGCAGAGCCTGTT |
| EPCAM | Human | GACTTTTGCCGCAGCTCAGGA | AGCAGTTTACGGCCAGCTTGT |
| MUC1 | Human | CTGTCACTGCCGCCGAAGA | CGTGCCCCTACAAGTTGGCA |
| KI67 | Human | CTACGGATTATACCTGGCCTTCC | AGGAAGCTGGATACGGATGTCA |
| Vimentin | Human | CGGGAGAAATTGCAGGAGG | TGCTGTTCCTGAATCTGAGC |
| Albumin | Human | CTGCCTGCCTGTTGCCAAAGC | GGCAAGGTCCGCCCTGTCATC |
| CYP3a4 | Human | AGCAAAGAGCAACACAGAGCTGAA | CAGAGGTGTGGGCCCTGGAAT |
| HNF4α | Human | GTACTCCTGCAGATTTAGCC | CTGTCCTCATAGCTTGACCT |
| ITGB1 | Human | GGACGCCGCGCGGAAAAGAT | CACCCACAATTTGGCCCTGCT |
| ITGA5 | Human | AGGGTCGGGGGCTTCAACTTA | GAGCGGCAGGGTGCATACTC |
